# The Ecology of Plant Chemistry and Multi-Species Interactions in Diversified Agroecosystems

**DOI:** 10.3389/fpls.2018.01713

**Published:** 2018-11-22

**Authors:** Rodolfo F. Silva, Gabriela B. P. Rabeschini, Giovanna L. R. Peinado, Leandro G. Cosmo, Luiz H. G. Rezende, Rafael K. Murayama, Martín Pareja

**Affiliations:** ^1^Programa de Pós-Graduação em Ecologia, Instituto de Biologia, Universidade Estadual de Campinas, Campinas, Brazil; ^2^Instituto de Biologia, Universidade Estadual de Campinas, Campinas, Brazil; ^3^Departamento de Biologia Animal, Instituto de Biologia, Universidade Estadual de Campinas, Campinas, Brazil

**Keywords:** volatile organic compounds, indirect interactions, ecosystem services, biological control, pollination, secondary plant chemistry, induced plant responses, agroecosystems

## Abstract

Over the past few years, our knowledge of how ecological interactions shape the structure and dynamics of natural communities has rapidly advanced. Plant chemical traits play key roles in these processes because they mediate a diverse range of direct and indirect interactions in a community-wide context. Many chemically mediated interactions have been extensively studied in industrial cropping systems, and thus have focused on simplified, pairwise and linear interactions that rarely incorporate a community perspective. A contrasting approach considers the agroecosystem as a functioning whole, in which food production occurs. It offers an opportunity to better understand how plant chemical traits mediate complex interactions which can enhance or hinder ecosystem functions. In this paper, we argue that studying chemically mediated interactions in agroecosystems is essential to comprehend how agroecosystem services emerge and how they can be guaranteed through ecosystem management. First, we discuss how plant chemical traits affect and are affected by ecological interactions. We then explore research questions and future directions on how studying chemical mediation in complex agroecosystems can help us understand the emergence and management of ecosystem services, specifically biological control and pollination.

## Introduction

It is well known that plant mediated interactions strongly influence the structure of natural communities (Connell and Slatyer, 1977; Roughgarden and Diamond, 1986). Resource competition (Connell, 1983; Schoener, 1983), allelopathy (Rice, 1984; Williamson, 1990) and facilitation (Hunter and Aarssen, 1988; Callaway, 1995) affect plant community organization, while plant mutualistic and antagonistic interactions with other organisms may structure communities (Van Zandt and Agrawal, 2004; Lewinsohn et al., 2006; Vizentin-Bugoni et al., 2014; Bergamo et al., 2017). Such interactions occur either by direct effects, when a plant trait affects the physiology or behavior of another organism; or by indirect effects, when an interaction with the plant has knock-on effects on a third organism not initially involved in this interaction (Ohgushi et al., 2007). Therefore, plant mediated interactions potentially link organisms of different trophic levels and add complexity within community interactions (Utsumi et al., 2010).

Plant-mediated interactions have been studied in agricultural systems due to the impact that herbivores, pathogens and pollinators have on plant production, and the mechanisms behind plant resistance, tritrophic interactions and pollinator attraction are well known (Agrawal and Rutter, 1998; Gatehouse, 2002; Klein et al., 2007; Schiestl, 2015). However, most studies have focused on commodity crops produced in large-scale monocultures. In Neotropical systems, there is surprisingly little focus on smallholder production systems (Pinto-Zevallos et al., 2016; Pinto-Zevallos et al., 2018), while soybean, maize and sugarcane are extensively studied (e.g., Peñaflor et al., 2011, 2017; Michereff et al., 2015). However, staple and horticultural crops are produced overwhelmingly by smallholders (Food and Agriculture Organization [FAO] et al., 2015) who often use intercropping and diversification practices (Altieri et al., 2012). These alternative practices are applied to manage agroecosystems based on ecological knowledge for ecosystem function improvement(Lewis et al., 1997; Vandermeer et al., 2010).

The integration of knowledge from chemical ecology into agroecology has been slow in the last decades, with a few notable exceptions (Cook et al., 2007; Khan et al., 2008, 2014). Chemical ecology can provide mechanistic knowledge of plant mediated interactions, which is essential due to the ecological complexity of agroecosystems. Chemical traits are important in mediating plant interactions because: (1) the high diversity of chemical compounds may confer unique combinations of primary and secondary compounds to species and individual plants (Krischik and Denno, 1989; Hartmann, 1996); (2) their expression is dynamic, changing with plant ontogeny and environmental cues (Barton and Koricheva, 2010; Quintero and Bowers, 2012); (3) they are subject to significant spatial and temporal variation (Feeny, 1970; Edwards et al., 1990; Okolie and Obasi, 1993); and (4) chemical perception is the dominant sensory mode in arthropods and microorganisms; these are the most abundant groups of organisms that interact with plants (de Bruyne and Baker, 2008; Bruce and Pickett, 2011; Junker and Tholl, 2013). Therefore, the plant chemical profile is a dynamic and multifunctional trait involved in a great diversity of interactions (Strauss and Agrawal, 1999; Arnason et al., 2004; Jones and Agrawal, 2016).

When secondary metabolites produced by plants are perceived by other organisms they acquire ecological functions and become subjected to selective pressures (Parachnowitsch and Manson, 2015; Jones and Agrawal, 2016; Petschenka and Agrawal, 2016). Some of these ecological functions are frequently explored in the literature, such as resistance against herbivory and mutualistic interactions (Strauss and Agrawal, 1999; Pichersky and Gershenzon, 2002; Heil and Karban, 2010; Jamieson et al., 2017). Following plant stress, there can be induced changes in plant traits like extrafloral nectar and secondary metabolites, which are tightly linked to other interactions established by plants (Agrawal and Rutter, 1998; Kessler and Baldwin, 2004; Heil, 2008). In particular, herbivore-induced volatile organic compounds (HI-VOCs) are involved in many interactions due to their ease of dispersal and perception by other organisms (Baldwin, 2010; Bruce and Pickett, 2011; McCormick et al., 2012). VOCs are detected by the emitter plants themselves, herbivores, predators, pathogens, parasitoids, hyperparasitoids, soil microorganisms, other plants and pollinators (Kessler and Halitschke, 2009; Junker and Tholl, 2013; Heil, 2014; Jamieson et al., 2017).

The fact that plant chemical traits such as VOCs have many functional roles makes them candidates for research and technology for agriculture. However, we still lack knowledge about the ecological consequences of the manipulation of plant chemical traits in a multitrophic and evolutionary context. Experimental manipulation of HI-VOCs emission by plants in the field, for example, is usually planned considering predators and parasitoids of herbivores as target receivers of these signals (Kaplan, 2012). However, such signals can also be perceived by other organisms in the community that interact with the focal plant, such as pollinators and other herbivores (Halitschke et al., 2008; Kessler and Halitschke, 2009; Jamieson et al., 2017). These interactions are considered non-target effects of HI-VOC manipulation and could result in positive or negative impacts on plant fitness. Due to the complex interactions mediated by volatiles (Dicke and van Loon, 2000; Dicke and Baldwin, 2010), non-target effects should be considered when manipulating volatiles in an agricultural context. Integrating this perspective in agroecosystems may help managing plant chemical traits more efficiently and responsibly.

In this paper, we propose that studying chemically mediated interactions structured by VOCs and phytochemical diversity in agroecosystems is essential to understand how ecosystem functions emerge and can be enhanced in these systems. We explore broad research questions and future directions on how chemical ecology can be studied in diversified agroecosystems and discuss its applications in two of the most studied agroecosystem services: pollination and biological control. By highlighting these perspectives, we hope to help comprehend how chemical ecology can be integrated in agroecosystem management.

## Integration of Chemical Ecology in Diversified Agroecosystems

Ecological interactions are responsible for providing important agroecosystem services. A goal of chemical ecology should be to help us understand these interactions so that agroecosystems can be managed more efficiently. To achieve this goal, a rigorous application of ecological theory is necessary. It is also fundamental to investigate traditional diversified agroecosystems that have been developed over thousands of years. Traditional cropping systems hold substantial amounts of information that detailed ecological study can make available more generally. For this purpose, we highlight two general interlinked questions that we think will be relevant in guiding future research.

(1) What are the mechanisms that generate phytochemical diversity in agroecosystems? Agroecosystem diversification can affect phytochemical diversity through three major paths: (1) planned taxonomic diversity, i.e., the plants chosen for inclusion in the agroecosystem; (2) associated diversity, which comprises the organisms that spontaneously appear in the agroecosystem; and (3) changes in plant chemistry resulting from induced responses to environmental cues such as biotic interactions and abiotic stress. Understanding the relevance of each of these processes for chemically mediated interactions is fundamental if we want to comprehend the ecological processes supporting agroecosystem function. VOC-mediated plant-plant interactions are promising candidates for these studies since it is known that they affect plant growth and defense (Ninkovic et al., 2002, 2003). It is possible that future research might reveal that specific plant combinations are effective choices for intercropping due to the characteristics of their VOCs-mediated communication and the consequences these VOCs have on multitrophic interactions.
(2) How does plant chemistry help modulate the interaction web in diversified agroecosystems? Research that focuses on pairwise interactions and/or linear tritrophic chains could lead to underestimation of the impacts of plant chemistry on interaction webs (Ohgushi et al., 2007). Agroecosystems are complex communities, and their management may alter the structure of interaction webs through modifications in plant diversity and, as a consequence, changes in plant chemical traits available to mediate direct and indirect interactions. By incorporating a whole-system approach (Lewis et al., 1997) that explicitly treats the agroecosystem as a complex of interactions, we may begin to understand the web of chemically mediated information superimposed upon the web of ecological interactions. Therefore, we can comprehand how plant chemistry affects the entire web, not only individual interactions (Larue et al., 2016).

The two topics highlighted above emphasize the mechanisms that generate phytochemical diversity and how plant chemistry affects the entire web of interactions. An integration of these two aspects is required to comprehend the emergence of agroecosystem services. This approach could provide knowledge to improve the quality of agroecosystem services through more efficient ecosystem management (e.g., use of plants with attractive compounds for natural enemies and/or pollinators). We can therefore analyze how different agroecosystem management practices affect the community structure and the plant chemical traits specifically related to ecological processes that guarantee such services (Lewis et al., 1997). In the following sections, we apply these general questions to two relevant ecosystem services for food production: pollination and biological control (Box 1).

**BOX 1. Future directions for studies of chemical ecology in diversified agroecosystems.**

The perspectives discussed on how plant chemistry can mediate ecological interactions in a community-wide agroecosystem context lead us to propose a series of research questions to comprehend the emergence of agroecosystem services. We focus on the application of chemical ecology to the study of pollination and biological control.

(1) What are the relative roles of taxonomic diversity, biotic interactions and abiotic stress in generating phytochemical diversity in agroecosystems?
(2) How does phytochemical diversity of VOCs affect pollinator diversity, flower-pollinator interaction webs and plant reproductive output?
(3) Do VOC-mediated plant-plant interactions have an effect on floral biology and agricultural production?
(4) How does phytochemical variation resulting from taxonomic plant diversity, biotic interactions and abiotic stress affect the natural enemies of herbivores in agroecosystems?
(5) How do VOC-mediated plant-plant interactions make plants more resistant or susceptible to herbivores and affect the structure of interaction webs?
(6) Are there trade-offs between cascading effects of induced plant responses on biological control and pollination at the agroecosystem level?
(7) Can knowledge of phytochemical traits be applied to improve crop combinations?

### Pollination

Agroecosystem diversification, through management of flower strips, for example, has shown that increasing plant diversity is positively associated with the richness and abundance of pollinators, pollination, and fruit production (Nicholls and Altieri, 2013; Pereira et al., 2015; Isbell et al., 2017). Moreover, in agroecosystems, flowers are not presented against uniform, monospecific backgrounds, but against variable mosaics of plant odors. Therefore, identifying the role of different processes generating phytochemical variation is fundamental to understand chemical mediation of pollination in agroecosystems.

The landscape of VOCs can be influenced by plant diversity and also by biotic interactions. For example, herbivore-induced changes in plant physiology can affect flower odor (Pareja et al., 2012) and pollinator abundance and behavior (Lucas-Barbosa et al., 2011; Lucas-Barbosa, 2016; Glaum and Kessler, 2017). This happens because the volatile signaling of flower rewards is the most reliable trait pollinators use in selecting flowers, so that when floral volatiles are more distinctive and less variable, pollinators learn to associate them with floral rewards more efficiently (Wright and Schiestl, 2009). Thus, if plants in highly diverse systems suffer lower herbivory (Randlkofer et al., 2010), and damaged plants attract fewer pollinators (Lehtilä and Strauss, 1997; Kessler et al., 2011) plants grown in polyculture may benefit from a more effective attraction of their pollinators (Orford et al., 2016; Isbell et al., 2017).

Floral volatiles are also important in plant-plant communication. They are produced and emitted at higher rates than vegetative VOCs, with over than 1700 known compounds. These compounds can vary quantitatively and qualitatively between species or between floral states within a species (i.e., pollinated vs. unpollinated flowers emit different signals; Knudsen et al., 2006; Raguso, 2008; Ibrahim et al., 2010). A plant receiving VOC signals gains valuable information about its reproductive environment, including signals regarding whether neighboring plants are in anthesis, whether they are hetero- or conspecific, and whether pollinators are present. Detecting the flowering state of neighboring plants can affect the floral traits of receiver plants such as anthesis, floral rewards and floral volatiles (Caruso and Parachnowitsch, 2016). Plant-plant VOC-based interactions require further study and will likely improve the efficiency of plant combinations for pollination management in agroecosystems.

It is also important to ask how phytochemically diverse environments affect the structure of plant-pollinator interaction webs. Each plant has specific floral traits that promote pollinator visitation while limiting non-pollinating visitors, a mechanism known as floral filtering (Junker et al., 2010). VOCs play an important role in floral filtering and present signals associated with flower presence and rewards recognized by flower visitors (Raguso and Willis, 2002; Cunningham et al., 2004; Milet-Pinheiro et al., 2012). Floral odors also act as guides with spatial orientation gradients, depending on the distribution of emitting sources on the vertical stratification of the vegetation (Baldwin et al., 2006; Randlkofer et al., 2010). Thus, choosing plants with diverse architecture in terms of vegetative and reproductive structure creates heterogeneous signals that can be efficiently exploited by multiple pollinators (Farré-Armengol et al., 2013).

### Autonomous Biological Control

Researchers have applied chemical ecology primarily in the study of individual pest species focused on pairwise interactions (Rosenheim and Coll, 2008). However, work in diversified agroecosystems shows that top-down and bottom-up ecological interactions can limit the population growth of species that may become pests (Letourneau et al., 2011). This ecosystem service has been termed Autonomous Biological Control (Vandermeer, 2011), Ecological Pest Management (Shennan et al., 2005) and Environmental Pest Management (Coll and Wajnberg, 2017). Chemical ecology has enormous potential for understanding ecological interactions in diversified agroecosystem management and ensure effective biological control.

Plant chemistry influences pest incidence by affecting plant localization and selection by herbivores (Webster et al., 2008) or through indirect defense, by the attraction of predators and parasitoids that may limit herbivory from the top-down (Aljbory and Chen, 2018; Furlong et al., 2018). Damaged plants emit a blend of VOCs that can signal herbivore presence and recruit predators, parasitoids or entomopathogenic nematodes (Rasmann et al., 2005; Filgueiras et al., 2016). Besides VOCs, other plant chemical traits also function to increase predator permanence on plants, like sticky compounds present in glandular trichomes that facilitate the capture of herbivores by immobilizing them (Romero et al., 2008) and extrafloral nectar that serves as an alternative food resource and enhances the presence of predators in the community (Heil, 2014; Rezende et al., 2014).

Despite research focused on the behavior of individual predators and parasitoids in response to volatiles in the laboratory, their response to volatiles in diverse odor backgrounds remains to be studied (Aartsma et al., 2017). VOC signals influence predator and parasitoid search behavior by relaying information about the presence of herbivores, the existence of other food sources (e.g., nectar and pollen), and the presence of plants that provide natural enemy-free space (Rossbach et al., 2005; Meiners, 2016). VOCs thereby lead different predator and parasitoid species to ignore, avoid or prefer particular odor mixtures (Perfecto and Vet, 2003; Wäschke et al., 2013). Therefore, VOC diversity resulting from agroecosystem management can influence the structure of trophic webs and help regulate herbivory by affecting herbivore localization by predators and parasitoids.

Plant-plant communication mediated by volatiles has rarely been included in biocontrol studies (Glinwood et al., 2011). It is known that signals emitted by plants can be detected by neighboring plants and can activate defense production in the receiving plants (Kost and Heil, 2006; Heil, 2008; Ninkovic et al., 2013). Perception of HI-VOCs by plants can lead to changes in their own VOC profiles, increase extrafloral nectar production, and enhance the attraction of natural enemies, leading to a reduction in herbivore pressure on the receiving plant (Glinwood et al., 2011; Heil, 2014). Undamaged plants also emit chemical signals that change the growth patterns, biomass accumulation, and defense mechanisms in neighboring plants (Glinwood et al., 2011). This communication affects multitrophic interactions as plants responding to volatile signals emit compounds repellent to herbivores and/or attractive to the natural enemies of herbivores (Ninkovic et al., 2013; Vucetic et al., 2014).

Push-pull technology is an example of how chemical ecology has been integrated to small-scale farming to improve pest and weed management (Khan et al., 2016). An example of successful push-pull system is the planting of *Desmodium* spp. among crop plants to act as ‘push’ components by emitting VOCs repellent to stemborers. Simultaneously, Napier grass is planted surrounding the crop as ‘pull’ components to chemically attract egg laying stemborers. Additionally, allelopathic compounds emitted by *Desmodium* spp. prevent the emergence of *Striga* ssp., a parasitic plant of maize and sorghum (Khan et al., 2008). The success of push-pull systems demonstrates how knowledge-intensive agricultural management can increase crop production and promote ecological interactions. Approaches like these involve the integration of chemical ecology in agriculture and collaborations between farmers and scientists (Khan et al., 2016).

Push-pull studies have broken new ground on communication between plants and plants and the third trophic level in diversified agroecosystems. A major goal should be to understand whether specific plant chemical traits have consistent effects in enhancing biological control across systems. This could lead to predictions of the effects of plant combinations on pest incidence based on VOC profiles. This information can be used to plan taxonomic and interaction diversity in agroecosystems.

## Conclusion

Chemical ecology has long focused on providing technological products for industrial agriculture, but it is now beginning to be recognized that small-scale agroecological systems have great potential to sustainably guarantee food security, particularly to the most vulnerable populations (Chappell and LaValle, 2011; Kremen and Miles, 2012; Food and Agriculture Organization [FAO], 2015). Such small-scale agroecological systems can greatly benefit from the perspectives discussed here: chemical ecology can shed light in the mechanisms of ecological interactions in these agroecosystems and thus build a collective base of transferable knowledge. In the few cases in which this approach has been implemented, it has shown enormous success for food security (Khan et al., 2016). We believe that further work on the chemical ecology of local production systems could contribute both to increase knowledge of chemical mediation of multi-species interactions in complex systems and to the development of more resilient and equitable crop production systems.

## Author Contributions

All authors contributed equally to the conception, literature search, and writing of the manuscript.

## Conflict of Interest Statement

The authors declare that the research was conducted in the absence of any commercial or financial relationships that could be construed as a potential conflict of interest.
